# A Novel Approach to Quantify Time Series Differences of Gait Data Using Attractor Attributes

**DOI:** 10.1371/journal.pone.0071824

**Published:** 2013-08-07

**Authors:** Manfred M. Vieten, Aida Sehle, Randall L. Jensen

**Affiliations:** 1 Department of Sport Science, University of Konstanz, Konstanz, Germany; 2 Department Health Physical Education Recreation, Northern Michigan University, Marquette, Michigan, United States of America; University of Maribor, Slovenia

## Abstract

In this paper we introduce a new method to expressly use live/corporeal data in quantifying differences of time series data with an underlying limit cycle attractor; and apply it using an example of gait data. Our intention is to identify gait pattern differences between diverse situations and classify them on group and individual subject levels. First we approximated the limit cycle attractors, from which three measures were calculated: *δM* amounts to the difference between two attractors (a measure for the differences of two movements), *δD* computes the difference between the two associated deviations of the state vector away from the attractor (a measure for the change in movement variation), and *δF,* a combination of the previous two, is an index of the change. As an application we quantified these measures for walking on a treadmill under three different conditions: normal walking, dual task walking, and walking with additional weights at the ankle. The new method was able to successfully differentiate between the three walking conditions. Day to day repeatability, studied with repeated trials approximately one week apart, indicated excellent reliability for *δM* (ICC_ave_ > 0.73 with no differences across days; p > 0.05) and good reliability for *δD* (ICC_ave_  =  0.414 to 0.610 with no differences across days; p > 0.05). Based on the ability to detect differences in varying gait conditions and the good repeatability of the measures across days, the new method is recommended as an alternative to expensive and time consuming techniques of gait classification assessment. In particular, the new method is an easy to use diagnostic tool to quantify clinical changes in neurological patients.

## Introduction

Typically, conventional kinematic analysis of human gait derives a characteristic pattern for an individual from a few single stride cycles [1], [2]. These approaches are very common in clinical trials and in clinical practice; however, a major disadvantage of this method is the neglect of essential information that may be included in the dynamical sequences of multiple strides during continuous locomotion [2]. Hence, some researchers have used other methods to analyze gait data, e.g. methods of non-linear time series [3]. Especially common is an approach using a non-linear time series analysis where Taken’s [4] embedding theorem enables the reconstruction of the phase state and the calculation and analysis of the maximal Lyapunov exponent [5], [6] is performed. The estimation of local dynamic stability can then be estimated though the largest Lyapunov exponent [7]–[12]. Perc [13] has built on these techniques in his study of human gait.

Non-linear time series approaches seem to have an advantage over conventional ones. However, while results are significant at the group level; a rating for an individual does not seem possible. van Schooten et al. [14] confirmed that depending on the state space reconstruction, local dynamic stability can be detected reliably enough to assess differences on the group level. However, on the individual level, they concluded that local dynamic stability only measures substantial changes, “which might not be realistic”. Looking at the theory behind this type of analysis, it seems understandable where problems might arise. The Lyapunov exponent as a measure for stable or unstable attractors was developed to examine deterministic chaos, i.e. describing (mathematical) systems without random elements involved. Time series, and gait data specifically, do contain random elements, which make the calculation of the Lyapunov exponent a tricky endeavor. A straight forward calculation of the Lyapunov exponent [15] as in the case of classical deterministic chaos is not possible; instead, estimation procedures must be applied. Two popular algorithms are those of Wolf et al. [16] and Rosenstein et al.[17]. However, these algorithms also have shortcomings [18], [19], which lead to the discussed problems of stability estimates for individuals. Floquet theory [20] is advocated for “… the study of the stability of linear periodic systems in continuous time.” In this case as well, theory works best when applying it to classical deterministic systems.

Our original task was to find a diagnostic tool that allows quantification of changing conditions in neurological patients, namely quantifying fatigue in the Multiple Sclerosis (MS) patient. The rationale is that movement patterns of gait show changes, when fatigue sets in. Ensuring that fatigue is the only possible reason for a pattern change in combination with a change in local variability would make it possible for the first time to quantify fatigue. The problem is that walking quality/stability of MS patients is very diverse. Consequently, the stability of the gait does not tell anything about fatigue, but its change would. Unfortunately, to the best of our knowledge, there is no method available allowing quantification of the changes in the movement pattern that are precise enough to rate the severity of fatigue on an individual level. Our new approach seeks to fill this gap and present a way to measure and document changes within the responses of an individual subject, as well as between subjects.

A dynamic system can be described by its state vector [21], and its undisturbed movement characterized by its attractor. Attractors represent equilibrium regions in the geometric space that are formed by the relevant variables describing the undisturbed movement dynamics [22]. Attractors can differ greatly in complexity. They can be as simple as a fixed point attractor, manifest as a limit cycle attractor, quasi-periodic, or be a chaotic attractor [22]. An example of a fixed point attractor in 3D coordinate space is the lowest point of a half-sphere shaped bowl at which a rolling marble will eventually stop moving. More complicated systems, such as humans walking, do not settle towards a point, but rather towards a track, which can be multi-dimensional and e.g. in the case of treadmill walking, is a closed loop, or limit cycle [13]. The problem with real world systems is their complexity. Building a model from first principles could mean the state space dimension could be very large [23]. In such a case, writing the full state vector seems impossible. We were looking for an approach, which would be sensitive enough to judge the differences of movement on an individual level. This requires a practical approach for such a task; one which need not include the complete state vector and therefore, is not overly demanding in terms of measurement and analysis. A part of the state vector and the respective attractor should characterize the walking movement sufficiently without being too complex, yet without the lack of essential information. Any vector coordinate of a point on the human body can serve as part of the state vector, which can be compared to the respective parts of its attractor. Points at each ankle can establish a good option, since from the supporting leg to the swinging leg we have a long kinematic chain with a high degree of freedom. Hence the possible movement variability is very large and can be used to express substantial parts of the walking characteristics. For a further description of our method and its use, it will not be necessary to decipher the actual walking characteristics; it is sufficient to simply calculate the attractor in different situations and quantify its change.

In this work, we describe a novel method for calculating and interpreting attractor variations and the differences between the attractor and state vector when comparing two time series, with underlying limit cycle attractors. As an example we analyzed data from a treadmill study examining the influence a dual task (mental assignment) or an additional physical load has in causing changes in normal walking. It was hypothesized that the changes in gait pattern due to the dual task and/or the additional load can be captured using the changes in the attractor and the change of the attractor/state vector’s standard deviations, which are each calculated from the distance between the attractor and the state vector. While the purpose of the current study was not to directly examine gait, the index formed from these two variables can be used to detect the changes in gait pattern both at the individual level and at the group level. Finally, we tested the reliability of our new method.

## Methods

A time series is a sequence of data points, which can contain almost any kind of time sequenced data. When dealing with gait, coordinate positions, velocity, and acceleration data constitute meaningful choices. An effective description of the attractor can be given e.g. in coordinate, velocity or acceleration space [23], [24], which are connected via 
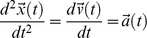
. For reasons that will become clear later, we have used the acceleration space.

The following method is valid for movements with an underlying limit cycle attractor. The time series data are treated according to the following simple model of the actual movement parameter: after transient oscillations have stabilized, the acceleration 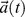 is governed by the attractor value 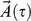 plus a fluctuating contribution 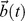 that varies around the attractor; this value “b(t)” is normally distributed with a zero mean.



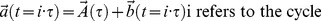
(1)


**Figure 1:**
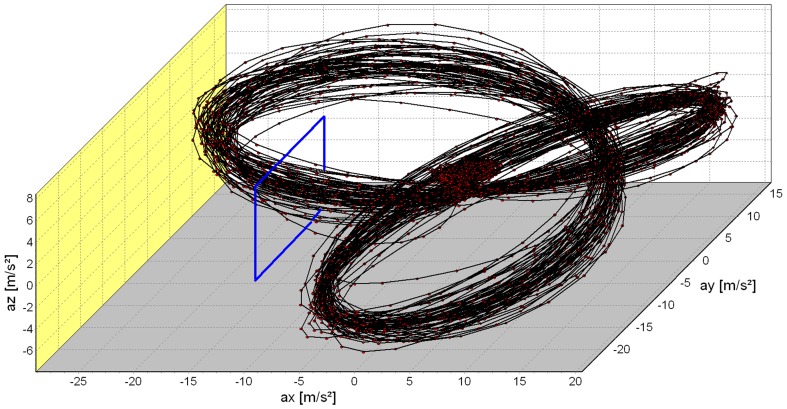
Three dimensional view of the acceleration data in m•s^−2^ illustrating the path of the state vector and cutting point to determine the start and end of a loop. Depicted is the side view of the area A (in blue) defining the start and the end of a cycle. All traces of the state vector must pass through and be perpendicular to the rectangle.



We measured two such parameters, the 3D acceleration (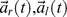) of the right and left ankles to allow detection of asymmetries in gait. The attractors’ 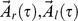
are approximated as the acceleration at time τ (ordering parameter of a complete cycle) averaged over all loops. 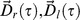 are the standard deviations describing the irregularities from the actual acceleration attractor. The start and the end of a loop are defined as the passing of 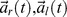 through a well-defined area A, as shown in Figure 1. With n being the number of measured cycles, the two expressions have the following form:

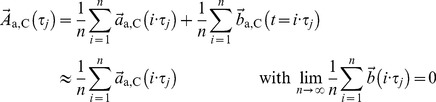
(2)




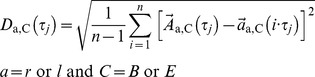
(3)


For the actual calculation of the attractors (equation (2)), the number of data points in cycles varies slightly. We term a data point of an attractor as valid, if the number of elements 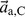 is at least 20% the number of elements of the first attractor point. This procedure is carried out via the software StatFree Version 7.0.3.1 (VietenDynamics, University of Konstanz, Germany; freely available on the Internet). Now the gait data can be compared at two different time intervals – index B: = begin, E: = end (e.g. one minute measuring at the beginning and at the end of treadmill walking). We define three parameters, wherein 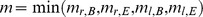 denotes the number of data points within the attractor with the fewest data points, v the walking speed and 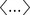 denotes average of the included expression: 
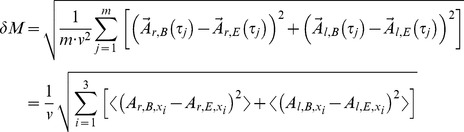
(4)

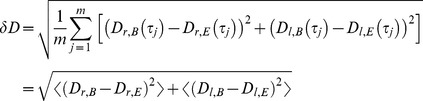
(5)

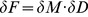
(6)
*δM* is the velocity normalized (see explanation below) square root of the squared average distance between two attractors, which can account for the change in the movement pattern of a person walking. *δD* identifies the fluctuation change of the state vectors around the two attractors, which quantifies a change in movement precision. The third definition (*δF*) is the product of the first two, which is an index being of interest in the event the changes affect the movement style and the movement quality simultaneously. *δM*, *δD*, and *δF* are invariant under rotation which easily can be shown. Let *δM’* be calculated in terms of a rotated coordinate system 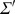. Any term of the form
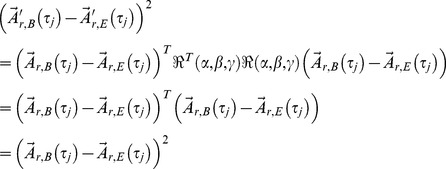
(7)


is invariant under rotation. Here the rotation matrix 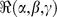 represents an arbitrary rotation and 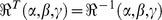 the transposed inverse matrix respectively. Both matrices can be functions of 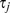
as well. All three expressions are combinations of those kinds of terms and therefore, are rotationally invariant.

Measurements can be done with a digitizing system giving the coordinates of a track, which permits approximating the attractor in coordinate space. However, using a kinematic tracking system requires a large degree of experimental effort and expense. A cheaper, simpler, and less work intensive system is an accelerometer. Mounted onto the human body, without using a gyroscope, it will not give acceleration data in the well-defined laboratory coordinate system, but since our three parameters are rotationally invariant this is not a hindrance. Furthermore, an additional argument for choosing acceleration over coordinate data is that the position on the treadmill results in some ambiguity; e.g. the subject’s position could be at the front of the treadmill throughout the first measurement and at the rear during the second measurement. This automatically would result in a huge *δM* even without being caused by a change in the walking style. This difference in measurement may be of interest if coordinate position is relevant in a study. Otherwise this variation can be removed by using the velocity instead of coordinate data as shown in the following equation.



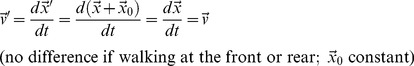
(8)


By taking the acceleration data instead of the velocity data, one is not even restricted to constant speed treadmill walking (see equation (9)).



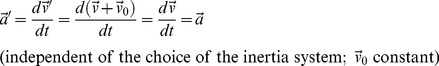
(9)


To differentiate coordinate data twice causes a substantial ratio of high frequency noise in the signal [25]. Therefore, a low pass filter must be applied when using data from a digitizing system. Temporary accelerometers are not free of such noise problems either and thus a low pass filter is required here as well. From these statements it is clear that the described method analyzes the low frequency information of our example time series. However, this is not a principal feature of our method. If data with a low noise ratio is available the full information content, with the high frequency part included, can be accessed.

A few remarks on normalization: *δM* as well as *δD* contains a factor 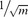, which accounts for the different time intervals and sampling frequencies of different measuring situations, equipment, and subjects. The factor 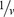 within *δM* is included to make results from measuring subjects at different walking speeds comparable. This can be understood in the following way:

The subject is walking at a speed 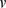. While a foot is on the ground/treadmill it has the relative velocity of zero. The foot in a swing phase must accelerate to make up for the time during stance. Hence, the average walking speed of one cycle is equal to the average velocity of the foot. 
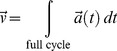
(10)


To compensate for different walking velocities we normalize to the walking/treadmill speed.
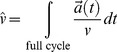
(11)


For a normalized walking speed the parameter 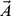 must be substituted by 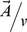. As a last step the average velocity 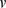 is moved to the front of the equation. Thus it might be possible to use our method in the case of a non-constant walking speed, making the measuring situation once again simpler. If possible, 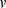 can be substituted by 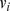 the average velocity of a cycle and the velocity would still be under the summation sign. However, we did not test this even more general procedure within the current study. In any case, *δM* and *δD* are abstract expressions and the dimensions are not of particular importance since they are not used for further calculations. However, we have used SI units to make sure the numbers can be compared; *δM* is given in s^−1^ while *δD* has the dimension m•s^−2^.

### Adaptation to a specific measuring situation and calibration

**Figure 2:**
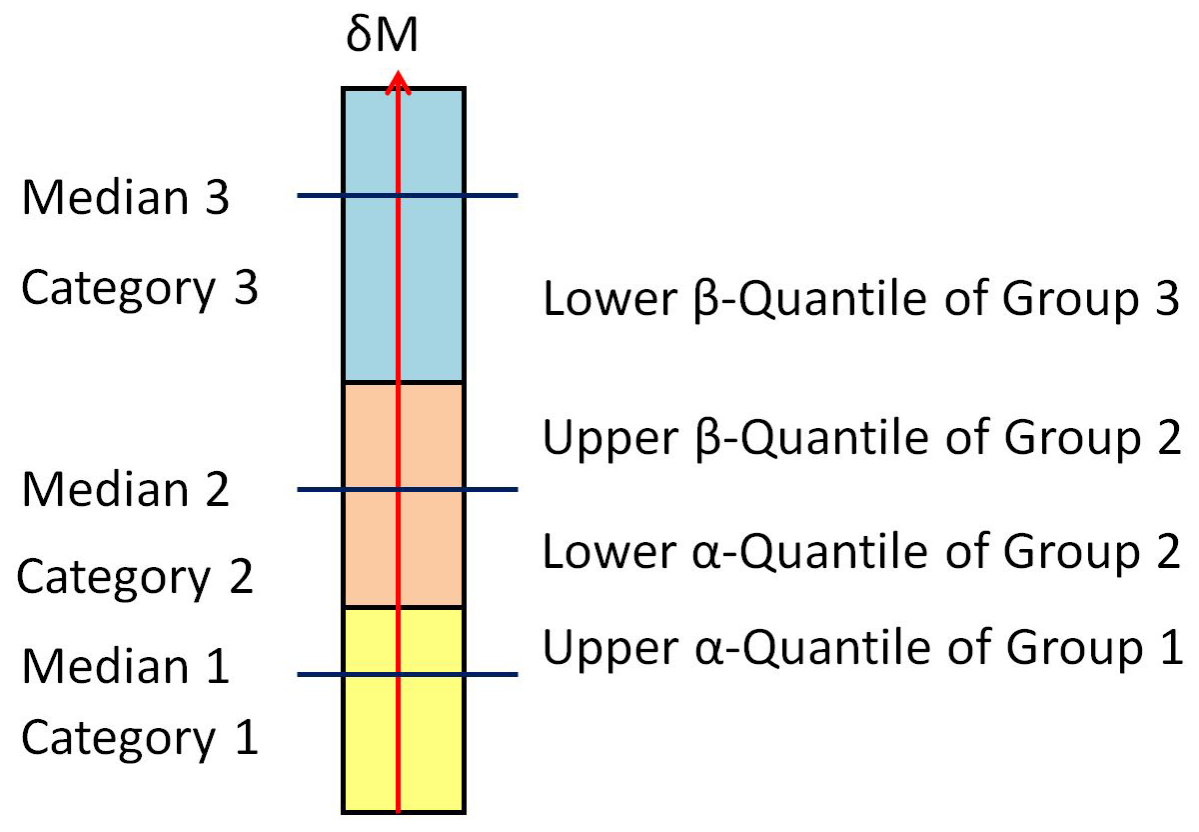
Illustration of example categories used to allow the creation of Quantiles from the medians of different groups.

*δM*, *δD* and *δF* represent relative results, which means a first measurement sets a baseline and the deviation between the base and the second measurement is calculated. This is a major difference compared with the calculation of the Lyapunov exponent that gives a kind of stability measure, an absolute quantity [7]–[12], [26]. However, for practical use the outcome still must be interpreted for both cases. For our method an interpretation schema can be established in two ways. If no known or appropriate classification is available, a given spectrum can be divided into subsections with increasing numbers denoting more crucial changes. If groups are definable, using well-known conventional methods [1], [2], medians of *δM*, *δD,* or *δF* are calculated for each group. While conventional methods are not sensitive enough to judge individuals, they do produce adequate group results. Also, by calculating the median, not the mean, we avoid the substantial influence of outliers. The group intervals are obtained by calculating the upper α-quantile of the group with the smaller median and the lower α-quantile of the group with the bigger median as consecutive non-overlapping areas. For the next two consecutive groups we calculate upper β-quantile and lower β-quantile connected with the next set of medians and so on for all adjacent groups (see Figure 2). These resultant numbers – e.g. 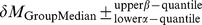 - serve as a calibration for our method. Once this procedure is done, individuals can be sorted into categories by calculating the appropriate *δM*, *δD* or *δF*.

### Method inherent aberration

As with all measurements, the outcome depends on the sensitivity and accuracy of the measuring equipment. In addition the method can cause extra deviations. This method’s inherent deviations from an exact numerical value are extremely small. However, there are two reasons for errors: 1) The first data point within a cycle is defined as the first measurement after passing the area A (Figure 1). These can be located within a volume 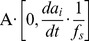, not directly on the area A. For this reason we do have a subtle dependence of the outcome on the definition of the cycle’s start, which can be decreased by raising the sampling frequency f_s_. 2) The attractor’s standard errors along the attractor path are dependent on the number of cycles analyzed. For each data point on the attractor path we have the real attractor 
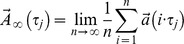
 located (one sigma probability) within an interval defined by 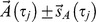 with 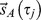 being the standard error. This induces the error margins for *δM,* which we calculate using the error propagation of independent variables. 
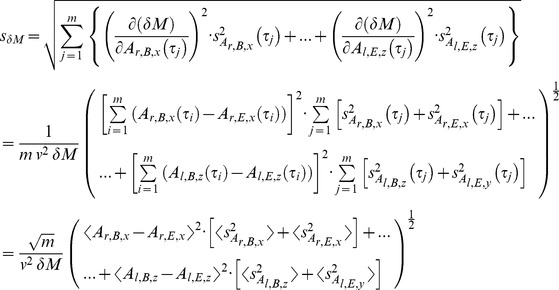
(12)


The complete expression consists of 6 terms (right and left side with three components each). The deviation of *δD* appears to depend via *D*(τ_j_) on the standard error 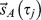 as well, but it does not. Formally the deviation is 
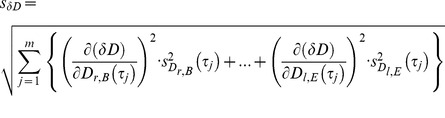
(13)


with 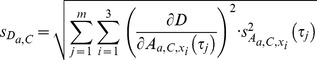
(14)and a = r or l and C = B or E.

First we calculate the derivative of *D*(τ_j_) in regard to one component of 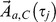


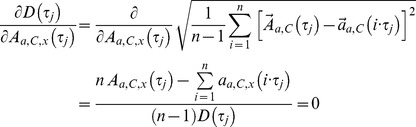
(15)


As a consequence s*_δD_* is identically zero 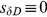
(16)since all derivatives have the same result. This might look strange, but it is a consequence of the definition of *D*, in which attractor inaccuracies cancel each other. *δF*’s deviation is simply
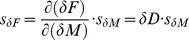
(17)


### Experimental procedures

Thirty healthy subjects (11 female, age: 31±11 years, height: 1.70±0.07 m, mass: 60±9 kg; 19 male, age: 29±11 years, height: 1.82±0.05 m, mass: 82±12 kg) participated in the study. The study protocol and informed consent process were approved by the local ethics committee of the University of Konstanz, Germany and was conducted in accordance with the Declaration of Helsinki. All subjects gave written informed consent according to this approval.

The participants performed a walking test on a treadmill under three different conditions: 1) five minutes walking without any load (N); 2) five minutes walking with an additional mental task (M); and 3) five minutes walking with a two kilogram weight on each ankle (W). Each of the combinations NM, NW and MW were done using equations (4) and (5) with B and E equal to N, M or W respectively. The treadmill speed was set to 1.39 m•s^−1^ and kept constant throughout the test. Thus one minute of walking was equivalent to about 60 cycles. Gait data were recorded while walking on the treadmill for one minute, for each subject, after each of the five minute conditions. This test was performed on two different days to check the repeatability.

Data were recorded using the RehaWatch 4.1.9.0 (HASOMED GmbH, Magdeburg, Germany). The equipment includes two inertial sensors, which were mounted on the lateral aspect of each ankle, and a data logger. Output data were internally corrected for data shift. The inertial sensors contain tri-axial accelerometers for measuring acceleration and tri-axial gyroscopes for measuring the angular velocity; the data of the latter were not used in this study.

### Data analysis

Analyses were limited to the acceleration data of the two foot sensors which were taken at a sampling rate of 500 Hz. We filtered the data with a 4.5 Hz low-pass filter [27] after performing a residual analysis [28] to find the optimal cutoff frequency. All statistical tests and numerical calculations were performed using StatFree, SPSS v19.0 (IBM, Armonk, NY, USA), and Microsoft Excel 2010 (Microsoft Office Professional Plus 2010). All group parameters were tested for normal distribution. Reliability estimates for *δM* and *δD* for each of the three conditions were performed by determining the day to day Intraclass Correlation Coefficient (ICC; 3, k) and differences between days via a paired t-test. Differences in normally distributed parameters between N and M, between N and W, as well as between M and W, were detected using a one-way Repeated Measures ANOVA. Bonferroni adjustment was applied to account for multiple comparisons, and the significance level for all statistical tests was set a priori to α = 0.005.

## Results

**Figure 3:**
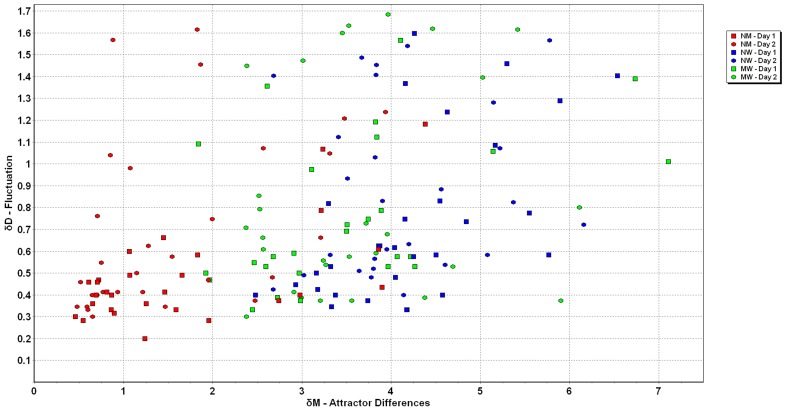
Scatterplot of *δM* and *δD* for the attractors in the three conditions. Note the clustering of data for each of the three conditions.

**Figure 4:**
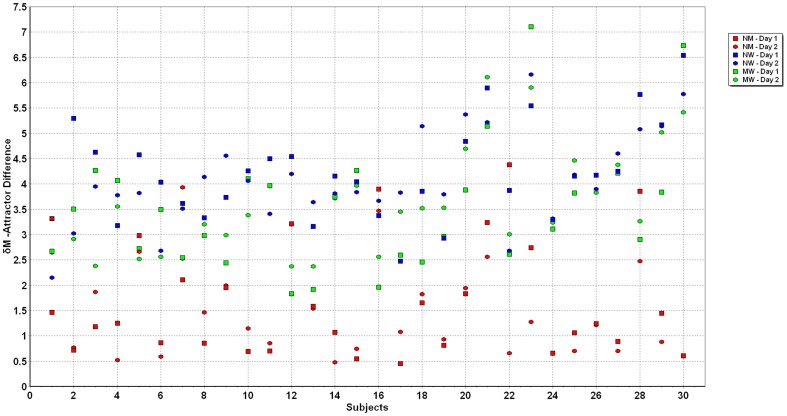
Individual subject values of *δM* for normal-mental, normal-weighted and mental-weighted walking on days 1 and 2.

**Figure 5:**
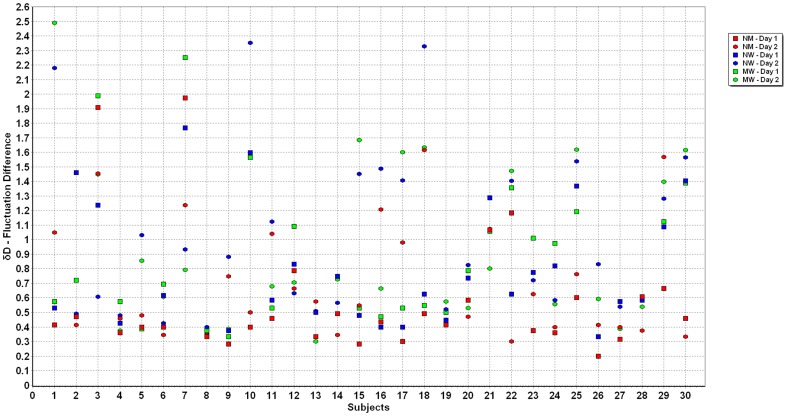
Individual subject values of *δD* for normal-mental, normal-weighted and mental-weighted walking on days 1 and 2.

The numerical values of all measurements of *δM* were between 0 and 7.1 s^−1^, while the variation of *δD* was between 0 and 2.5 m•s^−2^ (Figure 3). This graph shows the clustering of the data for each of the three conditions. Figures 4 and 5 illustrate the individuals’ data points for *δM* and *δD* for all three conditions on both days.

**Figure 6:**
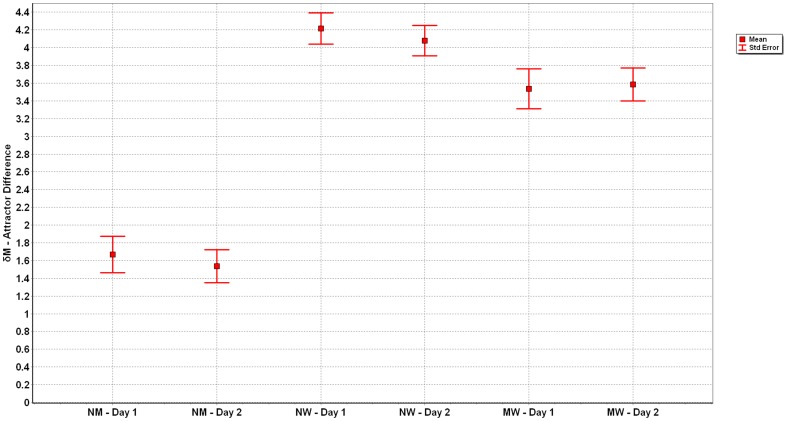
Means and Standard Error of *δM* for the three conditions across the two days. Significant differences occurred between all three tasks (p<0.001), but not within conditions.

**Figure 7:**
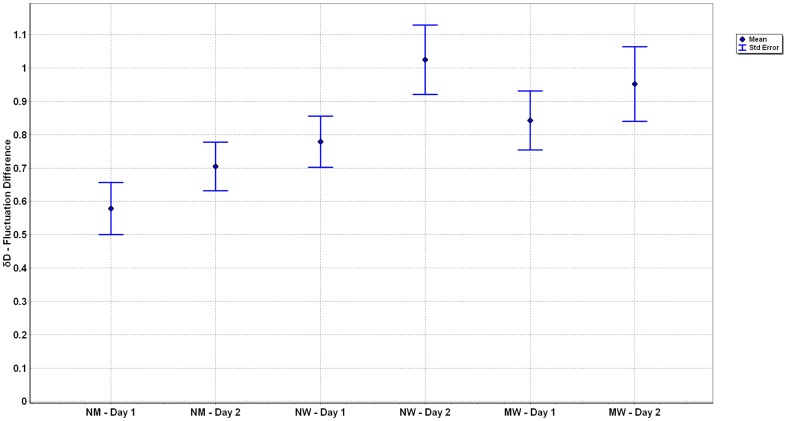
Illustration of the Means and Standard Error of *δD* for the three conditions across the two days. Significant differences occurred between all three tasks (p<0.001), but not within conditions.

The comparison of all three situations normal to mental, normal to weight, and mental to weight walking show significant differences (see Figure 6). The ANOVA revealed a significant main (p<0.001) effect for *δM*; with post hoc analyses showing that *δM* differed between all three tasks (p<0.001). Comparison of *δD* revealed a significant difference (p<0.001) between the three conditions. Post hoc analysis indicated that the fluctuation around the differences of normal to mental walking was less than either condition that included walking with weights on the ankles, i.e. normal to weighted and mental to weighted walking (see Figure 7).

Reliability estimates revealed that *δM* displayed excellent day to day reliability as indicated by the ICC and lack of differences (p > 0.05) between days (Table 1 and Figure 6). Day to day reliability of *δD* expressed via ICC was moderate to good and not different between days (p > 0.05) (Table 1 and Figure 7).

**Table 1:** Intraclass Correlation Coefficient (ICC) and the probability of a difference across days for *δM* and *δD* of normal walking without any load (N), walking with an additional mental task (M), and walking with two kilogram weights on each foot (W).

Measurements	ICC_ave_	Probability of Difference
*δM* N vs. M	0.732	p = 0.47
*δM* N vs. W	0.752	p = 0.38
*δM* M vs. W	0.881	p = 0.74
*δD* N vs. M	0.610	p = 0.13
*δD* N vs. W	0.414	p = 0.06
*δD* M vs. W	0.502	p = 0.36

The impact of different cutting conditions, defining the cycle’s start, were found to be smaller than 5% of the value of each parameter. We also calculated the error (equation (12)) caused by the attractor’s standard error and found s*_δM_* <0.05*δM*.

## Discussion

The purpose of this new method is to allow the easy quantification of differences between dynamic situations on the group and on the individual level. In the case of our example, differences between gait patterns under different constraints were determined. The constraints included normal walking, walking while performing a mental task (counting backwards by threes), and walking with weights added to the ankles. In the current study the gait pattern differed in all three conditions, i.e normal walking was different from mental and weighted walking, which also differed from each other. Previous studies have shown changes in gait patterns as a response to a cognitive or motor task while walking [29]–[33].

The estimation of reliability for the proposed methodology indicated that *δM* and *δD* are reliable and the results of one day can be replicated on a second day occurring approximately a week later. This is in agreement with the results of other studies. In a study with acceleration-based gait tests Senden et al. [34] found high repeatability in basic gait parameters such as step length, cadence, speed, and step time. Henriksen and coworkers [35] established the test–retest reliability of a trunk accelerometric gait test in healthy subjects. Gait parameters of step length, stride length, cadence and the mean acceleration were found to be stable. Kadaba et al. [36] investigated repeatability of gait data in normal adults at three times on three different days. They showed the gait parameters “are quite repeatable”; with good repeatability within a test day, but less so for measurements on different days. Recently, van Schooten et al. [14] also found reliability of local dynamic stability within a day was good, but was only moderate to poor between days.

While, *δD* displayed lower repeatability, the ICC_ave_ was still moderate to good ranging from 0.414 to 0.610, with no differences across days (p > 0.05). This is in agreement with Senden and colleagues [34] who also found less repeatability in the irregularity and asymmetry of gait measures.

An interesting point to note from the current study was the ability of the method to identify outlying values from one of the subjects who had different experiences between days. Subject 22 performed the mental task in a non-native language and commented afterwards that, “I was more nervous about getting the answers correct on the first day than the second day”. This subject displayed a much larger *δM* for the normal to mental walking on day 1 than on day 2 (see Figure 4). This instance, while anecdotal, suggests that when an individual displays characteristics that differ from the group, he/she will be classified into a different group by their *δM* and *δD*. Thus the sensitivity of *δM* and *δD* appears to allow the sorting of individuals into the various categories as explained in the methods section.

The described new method relies on thousands of data points for each subject and measurement. This makes for a strong statistical outcome. The attractor change *δM* and the fluctuation change *δD* for two different time intervals can then be reliably quantified at a high degree of accuracy. In our example study the error margins of *δM* and *δD* are in the range of 5%. Therefore, individuals can be rated precisely and categorization on a personal level is possible. The method is easy to apply, simple to use in the case of gait data acquisition, the analysis is uncomplicated, and results are sensitive and stable. Thus it appears that the new method could be useful in many situations, but may be most helpful in clinical settings where the low cost of accelerometers and minimal amount of data processing required would be desired.

A comparison between the well-established non-linear methods calculating the Lyapunov exponent or the Floquet multiplier shows big differences compared to our method. Lyapunov exponent and the Floquet multiplier were developed for the analysis of classical deterministic systems. Those methods allow deep insight into the mathematical working of dynamical systems, an ability that our method lacks completely. The application of established non-linear methods to data obtained from experiments is not without problems. To be able to handle more or less noisy data, algorithms have been developed (for a comprehensive overview see [15]), but the liability of these algorithms is still not completely assured [18]. On the other hand our method is developed to handle real live data of processes with an underlying limit cycle attractor only. The two main parameters in our method calculate the velocity normalized mean distance between limit cycle attractors (*δM*) and the change of the variation, which is the deviation of actual movement compared to the attractor (*δD*). The simplicity of our method and its stability allows quantification of factors responsible for changing movement pattern and movement variation as in the clinical example mentioned above.

In a clinical setting a central object is to diagnose functional problems which are reflected as walking abnormalities or changes in gait patterns between two different conditions. For example, fatigue is a common and frequently disabling symptom of Multiple Sclerosis with negative effects on normal activities of daily life [37]. Despite the high incidence of fatigue, there are no objective measures for assessing motor fatigue in Multiple Sclerosis. With our new method we can quantify the changes in gait patterns between two conditions: at the beginning and at the end (under full exertion) of the treadmill walking for each individual. If the change in a patient exceeds the threshold value – which we can define using the results of a control group – then the individual can be classified as a patient with fatigue symptom. In addition it could be used in other neurological conditions, such as Parkinson’s Disease, stroke, trauma, etc. Thus, the proposed method does not judge the quality of the movement, but allows quantification of the changes in gait pattern between two different conditions and hence gauges the acuteness of a neurological detraction. We therefore recommend this novel method as a tool to use in clinical studies and in the clinical practice.
